# Editorial

**Published:** 1876-07

**Authors:** 


### Editorial.

### CELLULOID.

"Is the use of celluloid for dental purposes an infringement on the Cumming's patent?"

According to justice and fact, *No!*

According to the Goodyear Dental Vulcanite Company, *Yes!* And just so long as the latter can browbeat and stamp down the former, just so long will that Company, through the machinations of its wiley, unscrupulous agents, prevail. A large number of the dentists of the country having been so much annoyed and harrassed by exactions for the use of rubber for artificial dentures, have abandoned that material and taken up celluloid, which they find to be equal in every respect to rubber, and in some particulars, its superior. As soon as this became apparent, the Rubber Company made claim upon celluloid, and unfortunately some in the profession are yielding to this claim.

The Celluloid Company have been criticised for what to some seemed to be neglect on their part in reference to the interests of those, in the dental profession at least, who were using their productions. There has been a general impression that all who would use celluloid would be protected in their right to do so by the Celluloid Company. It seems that from some quarter the impression has gone abroad that the Celluloid Company would not make any defense nor take up a case in which the question of the use of rubber was at all involved.

Such an impression or statement it seems, was never authorized by the company in any way whatever, as will be seen by the following statement from Mr. M. Lefferts, the President of the Company.

He says: "We are not willing to defend a suit where rubber alone is in question, but we *are* willing when celluloid is properly involved. Of course you will readily perceive the enormus burthen which is thus thrown upon our shoulders by Bacon's suing for rubber—and which he alone has the right to do—and incidently bringing before the master after its reference from the courts, the question of celluloid, and thus surreptitiously getting the master to pass upon the question and then parading it before the dentists at large as a suit in his favor. There have been three such cases, neither of which were we made aware of until after it was too late for us to interfere; but any one knows that such a decision has no binding effect outside of the persons against whom the immediate judgment was rendered. The master to whom the question of damages is referred has no right whatever to pass upon the question of celluloid, and such a question has no legal bearing upon the settlement of the question at issue, whether celluloid is an infringement of the Cumming's patent."

The following from Mr. M. C. Lefferts, the Treasurer of the Celluloid Company, will give some idea of what that company is doing in behalf of the dentists. He says: "I will briefly state what we have done in regard to all suits brought in May term. As we found many dentists who were notified of suit, either did not send us notices at all, or else too late to be of any use, either through negligence or design; we personally procured from all the district courts in the U. S. a complete return of all the parties sued by the G. D. V. Co., where cases were returnable May 1st. In all of these cases our attorneys have appeared and taken all necessary steps, and we shall follow the same course for all returnable June 1st. * * * It is improbable that all of these cases will be brought to trial; the policy of the G. D. V. Co. seems to be to put them all off, and in spite of our repeated invitations to them to try the issue in any one of the cases, they have invariably declined, so that if we can succeed in forcing one of all the present suits—one hundred in number—to a trial, all the rest will probably be deferred, but in the mean time we hope to settle the matter by a suit we have brought against them in New York to restrain them by injunction from harrassing the dentists, which after vexatious delays is now being argued.

We have further sent out a lawyer to visit each place where suits have been brought, to advise with the dentists, and see that our attorneys in the different states are doing their duties properly." Thus it will be be seen that the Celluloid Co. desire to defend and protect the dentists in the use of celluloid, at least so far as they wish to be defended.

### THE DENTAL SOCIETY OF THE STATE OF NEW YORK.

The Dental Society of the State of New York, will hold its eighth annual meeting in the capitol at Albany, on Wednesday, Thursday and Friday, July 12th, 13th and 14th,

The meeting will commence at 10 o'clock a. m., on Wednesday, and adjourn on Friday. This plan is adopted not only in compliance with a standing resolution of the society, but also in response to a desire expressed by many members, that more time may be devoted to the discussions.

It is hoped that members will come fully prepared to discuss the subjects selected by the essayists, and to report "incidents of office practice," or the results of special lines of research and experiment, to which a portion of each day will be appropriated.

Members desiring to present voluntary papers will please notify O. E. Hill, 160 Clinton Street, Brooklyn, at as early a date as possible.

Demonstrations in Operative Dentistry will be given on Thursday morning, at which time an opportunity will be afforded to exhibit new appliances, materials, dental, goods, etc. Members and others desiring to place articles on exhibtion will please to communicate with W. F. Winnie of Albany, who will take charge of this arrangement.

The Board of Censors meet on Tuesday, July 11th, in the Capitol, for the examination of all candidates who present themselves for the diploma of the Society conferring the degree of "Master of Dental Surgery" (M. D. S.) Those desiring further information concerning this examination should address F. French, of Rochester, the Secretary of the Board.

Delegates from several State Societies are expected to be present, and visitors from kindred organizations in this or other States will be cordially received.

Members and others in attendance at this meeting are advised that accommodations are generously offered them by the proprietors of the Delavan House at reduced rates.

ORDER OF BUSINESS.

Calling the roll.
Reading the minutes of the last meeting.
Report of the Committee of Arrangements.
Reception of members and payment of dues.
President's address.
Report of Treasurer.
Report of Correspondent.
Report of Censors.
Report of Committee on By-Laws.
Report of Committee on Ethics.
Report of Committee on Publication.
Report of Committee on Business.
Reports of Special Committees.
Reports of District Societies.
Reports of Dental Colleges.
Reading of essays and discussions.
Miscellaneous and unfinished business.
Election of officers.
Appointnent of committees.
Adjournment.

SUBJECTS AND ESSAYISTS.

Exposed Pulps,—W. S. Elliott, Goshen.
Choice of Material in Filling Teeth,—S. B. Palmer, Syracuse.
Encyclopedia of Practical Dentistry,—B. R. M'Gregor, Rochester.
Inflammation,—L. F. Harvey, Buffalo.
Pus,—W. H. Atkinson, New York.
Violated Laws,—L. S. Straw, Newburgh.
Practical Hints,—C. E. Francis, New York.
Dental Pathology,—C. A. Marvin, Brooklyn.
Dental Societies,—H. A. Baker, Woodstock, Vt.

### NEW JERSEY STATE DENTAL SOCIETY.

The sixth annual meeting of the New Jersey State Dental Society will convene at Atlantic City, July 18th, at 10 a. m., and continue in session three days. A cordial invitation to meet with us is extended to the dental profession generally.

CHAS. A. MEEKER, Secretary.

### AMERICAN DENTAL ASSOCIATION.

As will be seen by the official notice on another page of this journal, this body meets in Philadelphia on Wednesday, Aug. 1st. A very large gathering of the profession, not only of this, but of other countries is anticipated on that occasion, and it will certainly be one of great interest.

Every society and dental college should be fully represented. It has been intimated that the time and place do not give promise for a successful meeting. That will depend upon the members. If they all go determined to make it a success it will be. In order to do this, the time designated for the session should be wholly devoted for that purpose, then after the adjournment let the Exposition or other matters receive undivided attention.

Owing to the rail road war in regard to fares from the West eastward, it is impossible to obtain anything definite in the way of excursion rates for those going to the Association, though we are assured that they will be as low as we have usually had on such occasions, and possibly lower. By the 15th of July we will be able, probably, to give definite information to any interested who will address us. Some enquiry has been made in regard to accommodations in Philadelphia for the members of the Association and their friends during the time of our meeting. No definite arrangement has yet been made, but as indications now are, there will be no difficulty in securing desirable accommodations in private houses in almost any part of the city, at from $9 to $12 per week, this including good rooms and two meals per day. We will have such arrangements by the 20th of July, as will enable all who wish to obtain good accommodations and at reasonable rates.

### AMERICAN DENTAL ASSOCIATION.

The sixteenth annual meeting of the American Dental Association will be held in accordance with a resolution adopted at Niagara in 1872 and and ratified in the same place in 1875, in the city of Philadelphia, commencing on Tuesday, the 1st day of August, 1876. The sessions will be held in the chapel of the church at the S. E. corner of Broad and Arch Streets, a central and convenient locality. It is expected that representatives of the profession from abroad will be present, and all delegates from local societies, as well as other members of the profession in the United States, and elsewhere, who may contemplate a visit to the Centennial Exhibition, are earnestly urged to arrange their plans with reference to attending this meeting, which it is hoped will be of unusual interest.

C. STODDARD SMITH,
Recording Secretary.

### AMERICAN ACADEMY OF DENTAL SCIENCE.

The ninth annual meeting of the American Academy of Dental Science will be held in Boston, on Monday, September 25th, 1876, at 10 o'clock a. m.

The annual address will be delivered at 2 p. m., by Dr, Robert Arthur, of Baltimore.

E. N. HARRIS,
Corresponding Secretary.

### KENTUCKY DENTAL SOCIETY.

It was our privilege to be present at the annual meeting of the Kentucky State Dental Society, recently held in Louisville.

A better meeting it has rarely been our fortune to attend. Some slight matters of discord were all removed, to the great satisfaction of all concerned, and the utmost harmony and good feeling prevailed throughout. The discussion of subjects presented was very interesting and instructive. We may, perhaps, give some of the thoughts presented, in the next number of the REGISTER, and also a paper on Therapeutics.

This society is in a better condition for thorough and efficient work than ever before.

### PERSONAL—EXPLANATORY.

Enquiries are frequently made as to the progress made in the preparation of the "History of Dentistry," which has been in charge of the editor of the REGISTER for about two years.

To all such enquirers I would say that the work proves to be of far greater magnitude than I at all conceived in the beginning, and it has been attended with labor and difficulties that require time to overcome. During the last year new duties and additional labor have fallen to my lot, so that the time that can be devoted to this work is much diminished. A large amount of material is in hand and being arranged. Nearly all in the profession, who have been solicited, have responded fully and well upon the subjects assigned them. A few yet have their matter in preparation, which will soon be forthcoming.

I hope to have the work ready for the press in a few months; in the meantime let all who can co-operate.

J. TAFT.

